# First Case of Bacteraemia Due to Carbapenem-Resistant *Bacteroides faecis*

**DOI:** 10.3390/antibiotics10030319

**Published:** 2021-03-19

**Authors:** Charlotte Kaeuffer, Tiffany Ruge, Laure Diancourt, Benoît Romain, Yvon Ruch, Benoît Jaulhac, Pierre H. Boyer

**Affiliations:** 1 Department of Infectious Disease, Strasbourg University Hospital, 67000 Strasbourg, France; charlotte.kaeuffer@chru-strasbourg.fr (C.K.); yvon.ruch@chru-strasbourg.fr (Y.R.); 2 Laboratory of Bacteriology, Hôpitaux Universitaires de Strasbourg, 67000 Strasbourg, France; tiffany.ruge@etu.unistra.fr (T.R.); jaulhac@unistra.fr (B.J.); 3 National Reference Center for Anaerobic Bacteria and Botulism, Institut Pasteur, 25-28 Rue du Docteur Roux, 75724 Paris, France; laure.diancourt@pasteur.fr; 4 Department of Digestive Surgery, Strasbourg University Hospital, 67000 Strasbourg, France; benoit.romain@chru-strasbourg.fr; 5 Institut de Bactériologie, Fédération de Médecine Translationnelle de Strasbourg, University of Strasbourg, 67000 Strasbourg, France; 6 CHRU Strasbourg, UR7290, ITI InnoVec, 3 Rue Koeberlé, 67000 Strasbourg, France

**Keywords:** bacteraemia, anaerobic bacteria, *Bacteroides fragilis* group, carbapenemase, antimicrobial resistance, *bla*_CfiA_

## Abstract

Multidrug resistant (MDR) bacteria are increasingly observed in nosocomial and community-acquired settings. Anaerobes are no exception to this rule, but there are fewer reports of MDR in the scientific literature on anaerobes than there are for other bacteria. In this short case report, we describe the first case of bacteraemia caused by a multidrug-resistant *Bacteroides faecis*, which produces a carbapenemase encoded by the *bla*_CfiA_ gene. This bacteraemia followed a digestive surgery operation. Surprisingly, these findings did not lead to a change in antibiotic therapy, probably because the patient’s clinical state had improved. Nevertheless this report calls for better knowledge of anaerobic bacteria and for a systematic antimicrobial stewardship procedure following bacteraemia.

## 1. Introduction

The World Health Organization recognizes antibiotic resistance as “one of the biggest threats to global health” [1]. Concerns have focused on aerobic and aero-anaerobic bacteria (especially on *Staphylococcus aureus*, *Enterobacterales*, *Enterococcus faecium*, *Pseudomonas aeruginosa* and *Acinetobacter baumannii*). Nevertheless, increasing antibiotic resistance is also readily observed in anaerobes, especially in bacteria of the *Bacteroides fragilis* group [2]. Indeed, resistance to several antibiotics is observed, including carbapenems. This latter resistance can be mediated by a metallo-β-lactamase, encoded by the *bla*_CfiA_ gene [3].

Anaerobic bacteria are an important cause of bloodstream infections. Bacteria of the *B. fragilis* group are more frequently isolated than other anaerobes (for example, Gram-negative bacilli, *Fusobacterium* spp. *Peptostreptococcus*, *Clostridium* spp.), but remain poorly known to clinicians [4]. As a result, this group may not be taken into account when choosing an antibiotic for treatment, resulting in a high degree of mortality, especially in bloodstream infections [5]. In this clinical case report, we describe the first case of bacteraemia caused by a carbapenemase producing *Bacteroides faecis*.

## 2. Case Report

**Figure 1:**
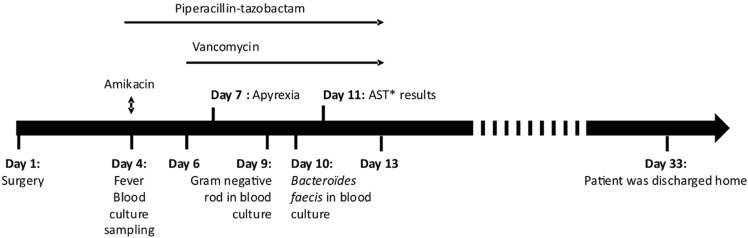
Chronology of the clinical case. (* AST: Antimicrobial Susceptibility Testing).

A 57-year-old man was admitted to hospital for an abdominoperineal resection and placement of an end colostomy. He had a history of perineal hidradenitis suppurativa and had been successively treated by multiple antibiotics (notably, ertapenem and doxycycline) and TNF-alpha inhibitors for years. Multiple fistulas and sphincter destruction later complicated his initial pathology, requiring several surgical treatments, including abdominoperineal amputation, followed by a perineal reconstruction using surgical flaps that was conducted on day 1 (Figure 1).

Post-operative history was marked by repeated vomiting, some of which was inhaled. An abscess on the laparotomy scar was also noted. Four days after this surgery, he was febrile which led to the sampling of two blood culture pairs. A blood sample for routine lab testing including blood count, C-reactive protein and liver and kidney function tests, was also taken with blood cultures. Blood analysis revealed an elevated C-reactive protein level at 215 mg/L (normal < 5mg/L) and white blood cell count at 12,980/mm^3^. He then received empirical antibiotic therapy based on piperacillin-tazobactam (4 g three times a day) combined with a dose of amikacin (1.4 g). Local care was provided, the scar abscess was flattened, and sessions of hyperbaric oxygen therapy were prescribed. Two days later, vancomycin (2 g per day) was added since he still had a fever. The fever sharply declined following vancomycin introduction.

While he was still apyretic, on day 9 after the surgery, one of the blood-culture bottles sampled during his febrile episode was detected as positive. Direct examination revealed a Gram-negative rod. The next day, *B. faecis* was identified using MALDI-TOF MS (Bruker Daltonic, Bremen, Germany). Antimicrobial susceptibility testing showed a resistance to beta-lactams, including carbapenems and clindamycin. This strain remained susceptible to metronidazole.

Since his clinical condition had improved, the antibiotic regimen was stopped after 10 days of piperacillin-tazobactam administration. He left hospital 4 weeks later. Figure 1 shows the chronology of the clinical case.

The *B. faecis* strain was sent to the French National Reference Centre for Anaerobic Bacteria and Botulism at the Pasteur Institute. Identification of *B. faecis* was confirmed by sequencing the 16s rDNA and of the *hsp60* genes. The *bla*_cfiA_ gene, encoding a metallo-β-lactamase which conferred the resistance to carbapenem, was detected. The additional antibiogram showed that the strain was susceptible to rifampicin, trimethoprim-sulfamethoxazole and moxifloxacin. Table 1 shows the minimum inhibitory concentrations (MIC) of different antimicrobial drugs, obtained after strain analysis.

One year later, the patient was still alive, and his medical condition had improved.

## 3. Discussion

Here, we report the first case of bacteraemia caused by carbapenem-resistant *B. faecis*. This resistance was mediated by a metallo-β-lactamase, encoded by the *bla*_CfiA_ gene.

Anaerobes are part of different normal flora, especially prevalent in the respiratory tract, in the vaginal cavity, on the skin, and foremost, in the digestive tract. In the latter, the Bacteroidetes phylum represents approximately 23% of the gut microbiota and includes several species of the *Bacteroides* genus, in particular, *B. fragilis* and *B. thetaiotaomicron* [6]. *B. faecis* belongs to the *B. fragilis* group and was isolated for the first time in 2010 by Kim et al. from human faeces [6]. Although anaerobes are commensal bacteria, they can cause severe infections, including bacteraemia [4]. They are isolated from 0.5 to 12% of positive blood cultures and members of the *B. fragilis* group are the most prevalent anaerobic bacteria involved in bacteraemia, with the primary disease usually being an intra-abdominal infection [4]. Up to now, three cases of *B. faecis* infections have been described (Table 2), all were due to intra-abdominal infections and strains were susceptible to piperacillin-tazobactam and carbapenems. However, *B. faecis* infections may be underdiagnosed due to identification difficulties and their possible misidentification as other *Bacteroides* species, such as *B. thetaiotaomicron* [6].

Carbapenem resistance is increasingly observed in bacteria of the *B. fragilis* group, notably strains that produce metallo-β-lactamases, encoded by the *cfiA* gene [2,9]. The expression of the *bla*_CfiA_ gene is linked to the presence of an insertion sequence which provides its promoter [10]. For this strain, it is clear that the *bla*_CfiA_ gene was expressed, since this *B. faecis* strain was resistant to imipenem and all beta-lactams, even those associated with beta-lactamase inhibitors. A study by Justesen et al. demonstrated that 10.2% of the *B. fragilis* group strains isolated in blood cultures from several Danish hospitals carried the *bla*_CfiA_ gene [11]. In contrast, a Hungarian study did not find strains carrying the *bla*_CfiA_ gene in blood culture isolates [2]. Other mechanisms causing carbapenem resistance have been described, such as penicillin-binding proteins modification [12] and over-expression of *bmeABC* pumps [13].

On the one hand, antibiotic resistance reduces the rate of appropriate empirical antibiotic therapy administration. On the other hand, without effective treatment, bacteraemia caused by anaerobes of the *B. fragilis* group has a high mortality rate. This rate varies according to the species isolated (24 to 31% for *B. fragilis*, 50% for *B. distasonis* and almost 100% for *B. thetaiotaomicron*) [4,6]. Moreover, an increase in mortality has been well documented in the literature in the absence of appropriate antibiotic therapy for *Bacteroides* spp. bacteraemia [5]. More surprisingly antimicrobial susceptibility testing results are not always taken into account in the antibiotherapy choice. As a consequence, an increase in mortality (from 18% when the antibiotic is adapted, to 55% when the antimicrobial is not adapted) was evidenced by Salonen et al. [5]. In this present case, the identification of a multi-drug resistant *B. faecis* responsible for the bloodstream infection did not result in a therapeutic change. This was probably because the patient’s clinical condition had improved after the empirical antibiotic administration. Despite the strain’s resistance to the administrated antibiotic treatments, the patient’s condition improved. We can hypothesize that this bacteraemia results from a digestive translocation with a very low inoculum, which is corroborated by the time of positivity of the blood culture which took a long time (5 days). In addition, the source of infection could have been controlled thanks to hyperbaric oxygen therapy sessions and to the surgical treatment of scar abscess.

Nevertheless, this clinical case illustrates the lack of medical knowledge on anaerobes, which calls for strong interactions between physicians, infectious disease specialists and clinical microbiologists in the form of an antimicrobial stewardship programs. Such strategies have demonstrated a decrease in mortality and a better management for *S. aureus* bacteraemia [14].

## 4. Conclusions

To our knowledge this is the first report of a bacteraemia caused by a carbapem-resistant, *bla*_CfiA_ producer *B. faecis*. This bacterium is rarely reported in humans, and may cause severe diseases. In this clinical case, digestive translocation probably caused the bacteraemia, which explains why the infectious situation was controlled without the use of efficient antibiotics.

Anaerobes are poorly known by physicians who not involved in infectious diseases, thus, increasing knowledge of these clinical cases is crucial to improve the treatment of patients.

## Figures and Tables

**Table 1:** Minimum inhibitory concentrations (MIC) and clinical categorization obtained for different antibiotics (Technic: Sensititre™ Anaerobe MIC Plate). EUCAST: European Committee on Antimicrobial Susceptibility Testing-CASFM: Comité de l’antibiogramme de la Société Française de Microbiologie.

Antibiotic	EUCAST Breakpoints	CASFM Breakpoints	MIC (mg/L)	Interpretation
Penicillin	0.25–0.5	-	>8	Resistant
Amoxicillin	0.5–2	-	>32	Resistant
Amoxicillin/clavulanic acid	4–8	4–8	>32	Resistant
Piperacillin/tazobactam	8–16	8–16	>128	Resistant
Piperacillin	16	-	>128	Resistant
Cefoxitin	-	-	>64	Resistant
Imipenem	2–4	2–4	>128	Resistant
Meropenem	2–8	2–8	>8	Resistant
Chloramphenicol	8	8	8	Susceptible-standard dosing regimen
Erythromycin	-	-	>128	Resistant
Clindamycin	4	4	>64	Resistant
Metronidazole	4	4	2	Susceptible-standard dosing regimen
Moxifloxacin	-	1–2	2	Susceptible-increased exposure
Tetracycline	-	-	>16	Resistant
Vancomycin	-	-	4	Resistant

**Table 2:** Cases of human infections due to *Bacteroides faecis* reported in the literature.

Reference	Type of Infection	Risk Factors	Resistance	Treatment	Outcome
Lee et al. 2015 [7]	Post-operative peritonitis	Sigmoid colon cancer	Piperacillin (SIE), cefoxitin, cefotetan	Piperacillin-tazobactam	Favorable
Lee et al. 2015 [7]	Bacteremia secondary to post-operative peritonitis	Rectal cancer	Piperacillin (SIE), cefoxitin, cefotetan	Piperacillin-tazobactam	Favorable
Garcia et al. 2016 [8]	Bacteremia secondary to colonic ischemia	Epicardic electrodes	Amoxicillin, piperacillin, clindamycin	Metronidazole	Death

## Data Availability

Data is contained within the article.
